# The incidence and prevalence of pterygium in South Korea: A 10-year population-based Korean cohort study

**DOI:** 10.1371/journal.pone.0171954

**Published:** 2017-03-27

**Authors:** Tyler Hyungtaek Rim, Min Jae Kang, Moonjung Choi, Kyoung Yul Seo, Sung Soo Kim

**Affiliations:** 1 Department of Ophthalmology, Severance Hospital, Institute of Vision Research, Yonsei University College of Medicine, Seoul, Korea; 2 Department of Ophthalmology, National Health Insurance Service Ilsan Hospital, Gyeonggi-do, Korea; 3 Yonsei Healthcare Big Data Based Knowledge Integration System Research Center, Yonsei University College of Medicine, Seoul, Korea; 4 Institute of Convergence Science, Yonsei University College of Medicine, Seoul, Korea; Chang Gung Memorial Hospital Kaohsiung Branch, TAIWAN

## Abstract

Although numerous population-based studies have reported the prevalences and risk factors for pterygium, information regarding the incidence of pterygium is scarce. This population-based cohort study aimed to evaluate the South Korean incidence and prevalence of pterygium. We retrospectively obtained data from a nationally representative sample of 1,116,364 South Koreans in the Korea National Health Insurance Service National Sample Cohort (NHIS-NSC). The associated sociodemographic factors were evaluated using multivariable Cox regression analysis, and the hazard ratios and confidence intervals were calculated. Pterygium was defined based on the Korean Classification of Diseases code, and surgically removed pterygium was defined as cases that required surgical removal. We identified 21,465 pterygium cases and 8,338 surgically removed pterygium cases during the study period. The overall incidences were 2.1 per 1,000 person-years for pterygium and 0.8 per 1,000 person-years for surgically removed pterygium. Among subjects who were ≥40 years old, the incidences were 4.3 per 1,000 person-years for pterygium and 1.7 per 1,000 person-years for surgically removed pterygium. The overall prevalences were 1.9% for pterygium and 0.6% for surgically removed pterygium, and the prevalences increased to 3.8% for pterygium and 1.4% for surgically removed pterygium among subjects who were ≥40 years old. The incidences of pterygium decreased according to year. The incidence and prevalence of pterygium were highest among 60–79-year-old individuals. Increasing age, female sex, and living in a relatively rural area were associated with increased risks of pterygium and surgically removed pterygium in the multivariable Cox regression analysis. Our analyses of South Korean national insurance claims data revealed a decreasing trend in the incidence of pterygium during the study period.

## Introduction

Pterygium is an abnormal overgrowth of the bulbar conjunctiva, and severe pterygium can cause astigmatism and visual disturbance [1, 2]. Pterygium also causes cosmetic problems. Although numerous population-based studies [3–8] have reported the prevalences and risk factors for pterygium, information regarding the incidence of pterygium is scarce. To the best of our knowledge, only two studies have documented the incidence of pterygium: the Barbados Eye Study [3] and the Beijing Eye Study [9]. Therefore, the present study aimed to investigate the incidence and prevalence of clinically diagnosed pterygium using a nationally representative sample of approximately 1,000,000 South Koreans from the National Health Insurance Service-National Sample Cohort 2002–2013 (NHIS-NSC 2002–2013).

## Methods

### Ethical considerations

This study adhered to the tenets of the Declaration of Helsinki, and the requirement for written informed consent was waived because of the retrospective design. This study’s design was approved by the institutional review board of the NHIS Ilsan Hospital (Gyeonggi-do, Korea).

### Database

A detailed profile of the NHIS-NSC 2002–2013 cohort has been previously published [10]. A nationally representative sample of approximately 1,000,000 South Koreans was selected using random sampling in 2002 (2.2% of the eligible Korean population, S1 Table). This cohort was followed for 12 years until 2013. During the follow-up period, the initial cohort size gradually decreased because of disqualifications from the NHIS (e.g., because of death or immigration). Therefore, to maintain the sample size over time, the cohort was refreshed each year by adding newborns, who were sampled using the 2.2% sampling rate to maintain the survey’s representative nature. This database provides detailed information regarding interventions, diagnostic codes, and personal demographic information in the reimbursement records from all kinds of medical facilities, which include hospitals, private clinics, and public centers in South Korea.

### Pterygium and sociodemographic factors

Cases of pterygium were defined based on an ophthalmologist examination and claim by the Korean Classification of Diseases code for pterygium (H110; corresponds to 372.4 from the International Classification of Diseases, Ninth Revision, Clinical Modification). Incident pterygium was defined based on the patient’s first diagnosis of pterygium, rather than a specific diagnosis involving the right and/or the left eye. Surgically removed pterygium was defined as patient cases with pterygium that required surgical removal more than once during the study period, based on the Korean Electronic Data Interchange codes (S5341 and S5342). The incident time of surgically removed pterygium was defined as the first diagnosis of pterygium.

The sociodemographic factors included age (0–9 years, 10–19 years, 20–29 years, 30–39 years, 40–49 years, 50–59 years, 60–69 years, 70–79 years, or ≥80 years), sex, residence (Seoul: Korea’s metropolitan capital city; a second area: the largest South Korean province; a third area: the second largest metropolitan city and two of the adjacent second-largest provinces; and a fourth area: all areas other than the previously mentioned geographical subdivisions), and household incomes. Korean citizens who are covered under NHIS are categorized as insured employees, insured self-employed individuals, and medical aid beneficiaries. Low-income individuals were defined as insured employees or insured self-employed individuals in the first to third income deciles, and medical aid beneficiaries. Middle-income individuals were defined as insured employees or insured self-employed individuals in the fourth to seventh income deciles, and high-income individuals were defined as insured employees or insured self-employed individuals in the eighth to tenth income deciles. All data were collected annually (e.g. a 49-year-old subject belonged to the 40–49 years age group in 2007 and to the 50–59 years age group in 2008).

### Incidence and prevalence of pterygium

To exclude subjects with chronic pterygium from the incidence calculation, we excluded patients who were diagnosed with pterygium in 2002 and 2003, and we included only patients who were first diagnosed with pterygium after 2003. The first visit to an ophthalmologist for pterygium was defined as the incident time. The annual incidence was calculated as persons who developed pterygium, based on the total at-risk population on January 1 of each year. Individuals without pterygium were followed-up starting on January 1, 2004, and the follow-up ended on the last day that the individual was covered by NHIS. For individuals with pterygium (incident cases), person-years were counted until the incident time. In cases of surgically removed pterygium, the incident time was defined as the first diagnosis of pterygium that was followed by surgical removal during the study period.

To include all subjects who had pterygium in the prevalence calculation, we included patients with chronic pterygium and calculated the prevalence from 2004. Annual prevalences were calculated based on the total NHIS-NSC population on January 1 of each year and all new cases that were diagnosed between January 1 and December 31.

### Statistical analysis

We calculated the incidences per 1,000 person-years and prevalences (%) per 100 persons with the 95% confidence intervals (CIs). Multivariable Cox proportional hazard regression analysis was performed to identify the risk factors that were associated with pterygium, and the results were expressed as hazard ratios (HRs) and 95% CIs. A two-sided p-value of <0.05 was considered statistically significant. All analyses were performed using Stata/MP software (version 14.0; StataCorp, College Station, TX, USA).

## Results

Table 1 shows the incidences of pterygium according to year (2004–2013) and according to the sociodemographic subgroups. A total of 21,465 persons (9,306 men and 12,159 women) were diagnosed with pterygium during the 10-year study period, and the average incidence was 2.1 cases (95% CI: 2.1–2.2 cases) per 1,000 person-years (1.8 per 1,000 person-years for men and 2.4 per 1,000 person-years for women). A total of 8,338 patients underwent surgical removal during the study period (3,553 men and 4,785 women). The incidence of surgically removed pterygium was 0.8 per 1,000 person-years (0.7 per 1,000 person-years for men and 1.0 per 1,000 person-years for women). The incidences of pterygium and surgically removed pterygium were highest among 60–69-year-old subjects (6.5 per 1,000 person-years and 2.8 per 1,000 person-years, respectively). The incidences of pterygium and surgically removed pterygium were higher in relatively rural areas, compared to the metropolitan city of Seoul. However, the incidences of pterygium and surgically removed pterygium were similar across the income levels.

**Table 1:** Incidence of Pterygium per 1,000 Person-years in South Korea.

	Pterygium				Surgically removed pterygium			
Variables	Person-years	No.	Incidence(95% CI)		Person-years	No.	Incidence(95% CI)	
**Year**								
2004	1,011,543	2,999	3.0	2.9–3.1	1,013,122	1,416	1.4	1.3–1.5
2005	1,008,888	2,908	2.9	2.8–3.0	1,010,436	1,335	1.3	1.3–1.4
2006	1,003,351	2,402	2.4	2.3–2.5	1,004,641	1,061	1.1	1.0–1.1
2007	1,037,893	2,260	2.2	2.1–2.3	1,039,100	898	0.9	0.8–0.9
2008	1,005,204	2,115	2.1	2.0–2.2	1,006,332	819	0.8	0.8–0.9
2009	1,006,376	2,018	2.0	1.9–2.1	1,007,422	751	0.7	0.7–0.8
2010	1,016,621	1,947	1.9	1.8–2.0	1,017,644	649	0.6	0.6–0.7
2011	986,491	1,931	2.0	1.9–2.0	987,530	631	0.6	0.6–0.7
2012	992,152	1,557	1.6	1.5–1.6	992,970	447	0.5	0.4–0.5
2013	991,866	1,328	1.3	1.3–1.4	992,552	331	0.3	0.3–0.4
Age (years)								
0–9	1,005,598	16	0.0	0.0–0.0	1,005,606	1	0.0	0.0–0.0
10–19	1,385,068	67	0.0	0.0–0.1	1,385,101	7	0.0	0.0–0.0
20–29	1,450,746	392	0.3	0.2–0.3	1,450,935	87	0.1	0.0–0.1
30–39	1,710,484	1,729	1.0	1.0–1.1	1,711,353	454	0.3	0.2–0.3
40–49	1,753,361	4,388	2.5	2.4–2.6	1,755,662	1,580	0.9	0.9–0.9
50–59	1,288,425	5,913	4.6	4.5–4.7	1,291,545	2,443	1.9	1.8–2.0
60–69	799,602	5,192	6.5	6.3–6.7	802,410	2,255	2.8	2.7–2.9
70–79	485,529	3,126	6.4	6.2–6.7	487,219	1,311	2.7	2.5–2.8
80	181,570	642	3.5	3.3–3.8	181,916	200	1.1	1.0–1.3
40	4,518,753	19,268	4.3	4.2–4.3	4,518,753	7,789	1.7	1.7–1.8
≥65	1,040,036	6,308	6.1	5.9–6.2	1,040,036	2,615	2.5	2.4–2.6
Sex								
Men	5,039,983	9,306	1.8	1.8–1.9	5,044,810	3,553	0.7	0.7–0.7
Women	5,020,400	12,159	2.4	2.4–2.5	5,026,937	4,785	1.0	0.9–1.0
Residence								
Seoul (metropolitan)	2,080,366	2,421	1.2	1.1–1.2	2,081,622	796	0.4	0.4–0.4
2nd area	2,322,724	3,479	1.5	1.4–1.5	2,324,560	1,201	0.5	0.5–0.5
3rd area	1,906,441	5,337	2.8	2.7–2.9	1,909,213	2,075	1.1	1.0–1.1
4th area	3,750,853	10,228	2.7	2.7–2.8	3,756,352	4,266	1.1	1.1–1.2
Household income								
Low	2,353,361	5,076	2.2	2.1–2.2	2,356,061	2,033	0.9	0.8–0.9
Middle	3,729,437	7,905	2.1	2.1–2.2	3,733,595	3,177	0.9	0.8–0.9
High	3,977,585	8,484	2.1	2.1–2.2	3,982,091	3,128	0.8	0.8–0.8
**Total**	10,060,383	21,465	2.1	2.1–2.2	10,071,747	8,338	0.8	0.8–0.8

CI: confidence interval.

Seoul: Korea’s metropolitan capital city; 2nd area: the largest South Korean province; 3rd area: the second largest metropolitan city and two of the adjacent second-largest provinces; 4th area: all areas other than the previously mentioned geographical subdivisions.

Table 2 shows that the prevalence of pterygium was 1.0% (95% CI: 0.9–1.0%) in 2004 and gradually increased to 2.6% (95% CI: 2.5–2.6%) in 2013. The period prevalences of pterygium and surgically removed pterygium were 1.9% (95% CI: 1.9–1.9%) and 0.6% (95% CI: 0.6–0.7%) between 2004 and 2013, respectively. There was an increasing trend in the prevalences of pterygium and surgically removed pterygium over time (1.0% and 0.3% in 2004, and 2.6% and 0.9% in 2013, respectively).

**Table 2:** Prevalence (%) of Pterygium Per 100 Persons in South Korea.

	Annual total population	Pterygium			Surgically removed pterygium		
Variables		No.	Prevalence(95% CI)		No.	Prevalence(95% CI)	
**Year**							
2004	1,016,580	9,694	1.0	0.9–1.0	2,723	0.3	0.3–0.3
2005	1,016,820	12,474	1.2	1.2–1.2	4,018	0.4	0.4–0.4
2006	1,002,005	14,611	1.5	1.4–1.5	5,018	0.5	0.5–0.5
2007	1,020,743	16,876	1.7	1.6–1.7	5,923	0.6	0.6–0.6
2008	1,000,785	18,234	1.8	1.8–1.8	6,528	0.7	0.6–0.7
2009	998,527	19,934	2.0	2.0–2.0	7,181	0.7	0.7–0.7
2010	1,002,031	21,704	2.2	2.1–2.2	7,806	0.8	0.8–0.8
2011	1,006,481	23,435	2.3	2.3–2.4	8,386	0.8	0.8–0.9
2012	1,011,123	24,785	2.5	2.4–2.5	8,763	0.9	0.8–0.9
2013	1,014,730	25,891	2.6	2.5–2.6	9,022	0.9	0.9–0.9
**Overall**							
All age groups	10,089,825	187,638	1.9	1.9–1.9	65,368	0.6	0.6–0.7
≥40 years	4,595,643	174,465	3.8	3.8–3.8	62,496	1.4	1.3–1.4
≥65 years	1,088,943	68,915	6.3	6.3–6.4	25,215	2.3	2.3–2.3

CI: confidence interval.

**Fig 1:**
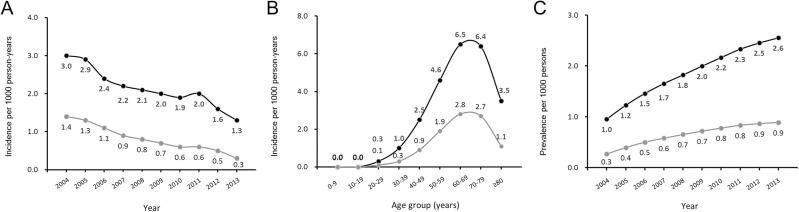
Incidence and Prevalence of Clinically Diagnosed Pterygium in South Korea. (A) Incidences of pterygium (black dot and lines) and surgically removed pterygium (gray dot and lines) per 1,000 person-years according to year. (B) Incidences per 1,000 person-years according to age group and (C) prevalence (%) according to year.

In the multivariable Cox regression model, the risk factors for pterygium and surgically removed pterygium were increasing age, female sex, living in a relatively rural area, and the middle income level (Table 3). Fig 1 shows the trends in the incidences and prevalences according to year and age group. The incidences of pterygium and surgically removed pterygium continuously decreased throughout the study period, although the prevalences of pterygium and surgically removed pterygium gradually increased over time. However, the magnitude of the prevalence increase was slightly reduced during the late study period.

**Table 3:** Sociodemographic Factors Associated with Pterygium and Surgically Removed Pterygium based on Multivariable Cox Regression (n = 1,116,364).

	Pterygium			Surgically removed pterygium		
Variables	HR (95% CI)		p-value	HR (95% CI)		p-value
Age group (year)						
0–9	0.01	0.01–0.02	<0.001	0.00	0.00–0.02	<0.001
10–19	0.05	0.04–0.06	<0.001	0.02	0.01–0.04	<0.001
20–29	0.27	0.24–0.30	<0.001	0.23	0.18–0.28	<0.001
30–39	1 (ref)			1 (ref)		
40–49	2.49	2.36–2.63	<0.001	3.46	3.11–3.84	<0.001
50–59	4.73	4.48–4.99	<0.001	7.67	6.93–8.47	<0.001
60–69	6.44	6.10–6.80	<0.001	10.79	9.75–11.93	<0.001
70–79	6.34	5.98–6.73	<0.001	10.52	9.45–11.71	<0.001
≥80	3.45	3.15–3.78	<0.001	4.42	3.74–5.22	<0.001
Sex						
Male	1 (ref)			1 (ref)		
Female	1.20	1.17–1.23	<0.001	1.22	1.16–1.27	<0.001
Residence						
Seoul (metropolitan)	1 (ref)			1 (ref)		
2nd area	1.42	1.35–1.49	<0.001	1.51	1.38–1.65	<0.001
3rd area	2.29	2.18–2.40	<0.001	2.66	2.45–2.88	<0.001
4th area	2.34	2.24–2.45	<0.001	2.93	2.71–3.16	<0.001
Household income						
Low	1 (ref)			1 (ref)		
Middle	1.22	1.18–1.27	<0.001	1.26	1.19–1.33	<0.001
High	1.15	1.11–1.19	<0.001	1.08	1.02–1.14	0.009

CI: confidence interval; HR: hazard ratio; ref.: reference group.

Seoul: Korea’s metropolitan capital city; 2nd area: the largest South Korean province; 3rd area: the second largest metropolitan city and two of the adjacent second-largest provinces; 4th area: all areas other than the previously mentioned geographical subdivisions.

## Discussion

In South Korea, the incidences of pterygium and surgically removed pterygium were 2.1 per 1,000 person-years and 0.8 per 1,000 person-years, respectively. In addition, the overall prevalences of pterygium and surgically removed pterygium were 1.9% and 0.6%, respectively, during 2004–2013. Although the incidences tended to decrease, the prevalences increased over time.

Two previous studies have evaluated the incidence of pterygium. The Barbados Eye Study included a predominantly black African Caribbean population (n = 1,888) in the West Indies, and revealed an incidence of 11.6% (n = 218) during the 9-year follow-up period [11]. This value corresponds to approximately 13 per 1,000 person-years among subjects who were ≥40 years old. The Beijing Eye Study included 2,628 participants and detected an incidence of 4.9% (n = 129) during the 10-year follow-up, which corresponds to an incidence of approximately 4.9 per 1,000 person-years among subjects who were ≥40 years old [9]. In the present study, the incidence of pterygium was 4.3 cases (95% CI: 4.2–4.3) per 1,000 person-years among subjects who were ≥40 years old. Thus, the incidence in the South Korean population is similar to that in the Beijing population, although it is approximately one-third of the incidence from the Barbados Eye Study. The similarity between the Chinese and Korean populations may be related to the shared race and similar genetic backgrounds. Furthermore, racial and climate differences may explain the higher incidence in the Barbados Eye Study, which was conducted in the topical Caribbean climate, where there are high levels of ultraviolet radiation. However, the present study used NHIS data, which is prone to selection bias because we could only identify patients with a confirmed and claimed diagnosis. Therefore, underestimation must be present, and it is possible that the South Korean incidence is higher than the Beijing incidence. Moreover, considering the decreasing trends in incidence over time that we observed, different study periods may provide different results, as the Barbados Eye Study was performed during 1988–2003 and the Beijing Eye Study was performed during 2001–2011.

Our group previously reported that the prevalence of pterygium was 8.9% (95% CI: 6.1–7.3%) among Korean subjects who were ≥40 years old, based on a nationwide population-based cross-sectional study (the 2008–2010 Korean National Health and Nutrition Examination Survey [KNHANES]) [8]. Another recent study using KNHANES 2008–2010 data also reported a similar value of 8.8% [12], and a study by the Epidemiologic Survey Committee of the Korean Ophthalmological Society (using KNHANES 2008–2009 data) also reported that the prevalence of pterygium was 8.9% [13]. In contrast, the prevalence in the present study was 3.8% (95% CI: 3.8–3.8%) among the same age group. However, the previous study actively screened participants for ophthalmological diseases, and it is likely that even very small pterygium (<1 mm) were included in the analysis. Furthermore, patients tend to not visit doctors during early disease stages, and the use of NHIS data in the present study indicates that the prevalence level is likely underestimated, and thus it may only represent the prevalence of symptomatic pterygium (i.e., pterygium that is large enough to prompt the individual to visit an ophthalmologist). Moreover, when our previous analysis was limited to pterygium with a size of >1 mm, the prevalence decreased to 5.1% (95% CI: 4.4–5.8%) among subjects who were ≥40 years old, which is similar to the result from the present study. Thus, the prevalence of clinically significant pterygium may be approximately 4–5% among South Koreans who are ≥40 years old.

Prevalences may vary according to the definition of pterygium, the use of a chart review or disease codes, and the population structure of the target country. Over the past century, decreasing fertility and mortality have led to the rapid aging of the South Korean population [12]. Therefore, based on the growing population of elderly South Koreans, it is impossible to directly compare our prevalence with those from other countries. However, we observed an explicit decreasing trend in the incidence and a gradual increasing trend in the prevalence. The decreasing incidence was an unexpected result, based on the fact that pterygium is associated with age and the Korean population is rapidly aging. However, over the past few decades, South Korea has experienced rapid socioeconomic growth, which may have improved public awareness regarding the important of eye protection (e.g., sunglasses during outdoor activities and safety glasses during work). We believe that this reduction may also be affected by the South Korean changes in the job structure and mobility. In South Korea, the proportions of farming, fishing, and forestry occupations were 29.0% in 1983, 17.5% in 1990, 10.0% in 2000, and 5.85% in 2010 (http://kostat.go.kr). Moreover, a national survey revealed that vitamin D deficiency was common among Korean wage workers, who were more likely to experience insufficient exposure to sunlight, and that study suggested that vitamin D management should be considered for Korean wage workers [13]. Thus, the environmental and occupational changes may influence the Korean incidence of pterygium, as ultraviolet light plays a key role in its pathogenesis, and office workers (less light exposure) is a growing subpopulation, compared to outdoor workers (more light exposure) [14]. We also found that the prevalence levels of pterygium and surgically removed pterygium in South Koreans exhibited relatively small increases during the late study period, which indicates that the prevalence levels may either reach a plateau or not significantly increase in the near future.

Pterygium was associated with increasing age, female sex, and living in a relatively rural area. However, our previous cross-sectional study revealed that pterygium tends to occur among elderly men who live in rural areas and have a history of lengthy sun exposure and low education level [8]. Moreover, our previous study revealed that pterygium appears to be more prevalent among people with a low sociodemographic status [8]. In the present study, we confirmed that increasing age and living in a relatively rural area were associated with an increased risk of pterygium. Furthermore, income level was not associated with pterygium in the present study, and this result is also consistent with the findings of our previous cross-sectional study [8]. However, we observed conflicting results regarding the association of pterygium and sex, as female sex was associated with this disease in the present study (vs. male sex in our previous study). This discrepancy might be caused by a higher detection rate among female South Koreans, as they are more likely to be concerned regarding cosmetic problems, compared to their male counterparts. We also observed that pterygium was associated with rural residency, which confirms the findings of previous major population-based studies [3, 4, 6, 7, 15–18].

### Study strengths and limitations

The present study’s greatest strength is its large sample size and prolonged follow-up (10,060,383 person-years and 10-year follow-up). However, as this study used an insurance database, there are some inevitable limitations. The most important limitation is that pterygium cases were identified using the Korean Classification of Diseases code, which may be less accurate than a medical chart review or cross-sectional evaluation. We were also unable to access important information regarding behavioral risk factors, such as smoking status and sun exposure. In addition, an assessment of the severity and duration of pterygium was not possible. Nevertheless, it is unlikely that a diagnosis of pterygium would be missed if it causes acute visual impairment, as South Korean patients have relatively good access to medical care and tertiary treatment centers [19]. Therefore, patients with pterygium are likely to visit an eye clinic based on self-recognition, which increases the likelihood of underestimating the incidences and prevalences. However, from a clinical perspective, symptomatic pterygium and surgically removed severe pterygium may be more relevant. Furthermore, the relatively long study period may increase the accuracy for calculating incidence and period prevalence, although there is likely a gap between disease occurrence and diagnosis. Moreover, the cohort’s ethnic composition may have contributed to the disparity between our data and those from other studies.

In conclusion, the present study evaluated the incidence of pterygium during a 10-year period using a nationwide claims database. Among the total population, the incidences of pterygium and surgically removed pterygium were 2.1 per 1,000 person-years and 0.8 per 1,000 person-years, respectively, and 4.3 per 1,000 person-years and 1.7 per 1,000 person-years among subjects who were ≥40 years old. We also observed a gradually decreasing trend in the incidence (from 3.0 per 1,000 person-years in 2004 to 1.3 per 1,000 person-years in 2013), despite the rapidly aging South Korean population. This information may provide insight regarding the incidence, prevalence, and future trends of pterygium in South Korea.

## Supporting information

S1 Table Cohort profile according to calendar year from 2004 to 2013. (PDF)
