# Supplementary material for: The incidence and prevalence of pterygium in South Korea: A 10-year population-based Korean cohort study
Source: PLoS One. 2017 Mar 27;12(3):e0171954. doi: 10.1371/journal.pone.0171954 (PMC5367680; doi:10.1371/journal.pone.0171954)
Supplement: S1 Table — (PDF) [file pone.0171954.s001.pdf]

S1 Table. Cohort Profile According to Calendar Year from 2004 to 2013.

	2004	2005	2006	2007	2008	2009	2010	2011	2012	2013
Age group										
0	9,320(0.9)	8,557(0.8)	7,872(0.8)	9,766(1.0)	9,393(0.9)	8,616(0.9)	9,032(0.9)	9,694(1.0)	9,851(1.0)	8,825(0.9)
1-4	43,571(4.3)	39,450(3.9)	35,720(3.6)	34,463(3.4)	34,044(3.4)	34,057(3.4)	34,174(3.4)	35,661(3.5)	36,055(3.6)	36,843(3.6)
5-9	69,802(6.9)	67,851(6.7)	64,410(6.4)	60,527(5.9)	55,151(5.5)	51,121(5.1)	46,314(4.6)	42,538(4.2)	42,745(4.2)	42,816(4.2)
10-14	73,939(7.3)	75,460(7.4)	73,799(7.4)	73,543(7.2)	70,027(7.0)	67,556(6.8)	65,658(6.6)	63,160(6.3)	58,652(5.8)	54,799(5.4)
15-19	66,457(6.5)	66,069(6.5)	66,775(6.7)	69,855(6.8)	69,998(7.0)	71,416(7.2)	72,987(7.3)	72,438(7.2)	71,260(7.0)	69,485(6.8)
20-24	79,641(7.8)	75,923(7.5)	69,764(7.0)	67,663(6.6)	63,529(6.3)	62,686(6.3)	62,514(6.2)	64,420(6.4)	66,975(6.6)	69,110(6.8)
25-29	78,614(7.7)	79,036(7.8)	78,547(7.8)	81,223(8.0)	80,465(8.0)	76,930(7.7)	73,424(7.3)	69,228(6.9)	65,376(6.5)	62,812(6.2)
30-34	92,742(9.1)	89,679(8.8)	84,119(8.4)	82,752(8.1)	77,216(7.7)	75,852(7.6)	76,338(7.6)	77,854(7.7)	78,621(7.8)	79,575(7.8)
35-39	91,928(9.0)	92,569(9.1)	93,107(9.3)	94,708(9.3)	91,844(9.2)	89,517(9.0)	86,649(8.6)	83,043(8.3)	80,239(7.9)	76,378(7.5)
40-44	91,891(9.0)	89,838(8.8)	85,940(8.6)	86,317(8.5)	86,834(8.7)	88,457(8.9)	89,462(8.9)	91,322(9.1)	91,837(9.1)	90,825(9.0)
45-49	81,809(8.0)	85,141(8.4)	87,975(8.8)	91,234(8.9)	89,447(8.9)	87,940(8.8)	86,394(8.6)	83,942(8.3)	83,334(8.2)	85,843(8.5)
50-54	56,731(5.6)	61,760(6.1)	66,331(6.6)	70,456(6.9)	73,322(7.3)	77,599(7.8)	81,356(8.1)	85,589(8.5)	87,599(8.7)	87,910(8.7)
55-59	46,314(4.6)	48,590(4.8)	48,391(4.8)	51,309(5.0)	51,088(5.1)	53,487(5.4)	58,594(5.8)	63,944(6.4)	67,116(6.6)	71,573(7.1)
60-64	43,019(4.2)	42,029(4.1)	40,710(4.1)	41,076(4.0)	41,773(4.2)	43,298(4.3)	45,700(4.6)	46,272(4.6)	48,438(4.8)	49,544(4.9)
65-69	35,554(3.5)	36,207(3.6)	37,216(3.7)	40,326(4.0)	39,770(4.0)	39,243(3.9)	38,660(3.9)	38,181(3.8)	38,220(3.8)	39,792(3.9)
70-74	24,518(2.4)	26,315(2.6)	27,738(2.8)	29,369(2.9)	29,925(3.0)	31,244(3.1)	32,110(3.2)	33,593(3.3)	36,145(3.6)	36,883(3.6)
75-79	15,614(1.5)	16,440(1.6)	17,146(1.7)	18,527(1.8)	18,849(1.9)	20,107(2.0)	21,759(2.2)	23,403(2.3)	24,741(2.4)	26,193(2.6)
80-84	9,326(0.9)	9,812(1.0)	9,969(1.0)	10,451(1.0)	10,517(1.1)	11,502(1.2)	12,383(1.2)	13,140(1.3)	14,169(1.4)	15,016(1.5)
≥85	5,790(0.6)	6,094(0.6)	6,476(0.6)	7,178(0.7)	7,593(0.8)	7,899(0.8)	8,523(0.9)	9,059(0.9)	9,750(1.0)	10,508(1.0)
Mean age (SD)	34.9(20.1)	35.4(20.1)	36.0(20.1)	36.5(20.1)	37.0(20.1)	37.6(20.1)	38.2(20.1)	38.7(20.1)	39.3(20.1)	39.8(20.2)
Sex										
Men	508,223(50.0)	508,317(50.0)	500,808(50.0)	510,009(50.0)	501,019(50.1)	499,689(50.0)	501,338(50.0)	503,428(50.0)	505,614(50.0)	507,289(50.0)
Women	508,357(50.0)	508,503(50.0)	501,197(50.0)	510,734(50.0)	499,766(49.9)	498,838(50.0)	500,693(50.0)	503,053(50.0)	505,509(50.0)	507,441(50.0)
Income level										
Low	226,114(22.2)	235,516(23.2)	231,669(23.1)	240,362(23.5)	239,572(23.9)	233,504(23.4)	239,525(23.9)	242,030(24.0)	220,175(21.8)	241,652(23.8)
Middle	390,192(38.4)	384,968(37.9)	378,412(37.8)	382,771(37.5)	371,657(37.1)	370,771(37.1)	367,424(36.7)	366,053(36.4)	350,343(34.6)	367,951(36.3)
High	400,274(39.4)	396,336(39.0)	391,924(39.1)	397,610(39.0)	389,556(38.9)	394,252(39.5)	395,082(39.4)	398,398(39.6)	440,605(43.6)	405,127(39.9)
Residence										
Seoul (metropolitan)	212,099(20.9)	211,125(20.8)	207,038(20.7)	214,064(21.0)	210,431(21.0)	209,028(20.9)	203,903(20.3)	202,595(20.1)	201,644(19.9)	200,395(19.7)
2nd area	218,495(21.5)	222,384(21.9)	221,526(22.1)	235,386(23.1)	234,656(23.4)	236,478(23.7)	233,374(23.3)	235,956(23.4)	239,361(23.7)	242,246(23.9)
3rd area	199,172(19.6)	197,881(19.5)	194,484(19.4)	192,284(18.8)	187,123(18.7)	185,476(18.6)	190,688(19.0)	191,209(19.0)	191,015(18.9)	191,178(18.8)
4th area	386,814(38.1)	385,430(37.9)	378,957(37.8)	379,009(37.1)	368,575(36.8)	367,545(36.8)	374,066(37.3)	376,721(37.4)	379,103(37.5)	380,911(37.5)
Total	1,016,580(100.0)	1,016,820(100.0)	1,002,005(100.0)	1,020,743(100.0)	1,000,785(100.0)	998,527(100.0)	1,002,031(100.0)	1,006,481(100.0)	1,011,123(100.0)	1,014,730(100.0)
